# A genome-wide CRISPR/Cas9 gene knockout screen identifies immunoglobulin superfamily DCC subclass member 4 as a key host factor that promotes influenza virus endocytosis

**DOI:** 10.1371/journal.ppat.1010141

**Published:** 2021-12-06

**Authors:** Yangming Song, Haixiang Huang, Yuzhen Hu, Jiwen Zhang, Fang Li, Xin Yin, Jianzhong Shi, Yanbing Li, Chengjun Li, Dongming Zhao, Hualan Chen

**Affiliations:** 1 College of Veterinary Medicine, Gansu Agricultural University, Lanzhou, People’s Republic of China; 2 State Key Laboratory of Veterinary Biotechnology, Harbin Veterinary Research Institute, Chinese Academy of Agricultural Sciences, Harbin, People’s Republic of China; The Ohio State University, UNITED STATES

## Abstract

Influenza virus infection is dependent on host cellular factors, and identification of these factors and their underlying mechanisms can provide important information for the development of strategies to inhibit viral infection. Here, we used a highly pathogenic H5N1 influenza virus to perform a genome-wide CRISPR/Cas9 gene knockout screen in human lung epithelial cells (A549 cells), and found that knockout of transmembrane protein immunoglobulin superfamily DCC subclass member 4 (IGDCC4) significantly reduced the replication of the virus in A549 cells. Further studies showed that IGDCC4 interacted with the viral hemagglutinin protein and facilitated virus internalization into host cells. Animal infection studies showed that replication of H5N1 virus in the nasal turbinates, lungs, and kidneys of IGDCC4-knockout mice was significantly lower than that in the corresponding organs of wild-type mice. Half of the IGDCC4-knockout mice survived a lethal H5N1 virus challenge, whereas all of the wild-type mice died within 11 days of infection. Our study identifies a novel host factor that promotes influenza virus infection by facilitating internalization and provides insights that will support the development of antiviral therapies.

## Author summary

Influenza virus infection is initiated by the attachment of the viral HA protein to sialic acid receptors on the host cell surface; most of the virus particles enter cells through clathrin-mediated endocytosis (CME). However, it is still largely unknown which protein(s) play(s) a role in transmitting the signal of viral binding across the plasma membrane to initiate CME. In this study, we found that the single-pass type I transmembrane protein immunoglobulin superfamily DCC subclass member 4 (IGDCC4) plays a key role in influenza virus internalization into host cells. IGDCC4 knockout dramatically increased the ability of mice to resist influenza virus infection. Our study provides insights that will support the development of therapies against influenza.

## Introduction

Influenza virus continuously evolves in nature and severely threatens both human and animal health with considerable impact on the global economy. Influenza virus causes human seasonal epidemics with 290,000 to 650,000 deaths annually worldwide, and caused serious pandemics with high morbidity and mortality in humans in 1918, 1957, 1968, and 2009 [1, 2]. Highly pathogenic avian influenza viruses, including H5 and H7 viruses, are always present in waterfowl and wild birds, and have sporadically crossed the species barrier to threaten public health [3–5]. H5N1 viruses have caused disease outbreaks in poultry and wild birds in more than 60 countries across three continents since 2003 [6–8], and over 850 human infections have been reported in 16 countries with a mortality rate of nearly 52% [9]. H7N9 viruses caused over 1,560 human infections in China and a mortality rate of 39% [10, 11].

Vaccination and antiviral drugs are effective ways to prevent influenza virus infection. Although vaccination provides adequate protection against seasonal influenza infection, the appearance of antigenic variants caused by antigen shift and antigen drift is faster than the development of new vaccines. At the beginning of an emerging pandemic, antiviral drugs will be on the frontline to prevent virus infection while a corresponding specific vaccine is developed [5]. There have been only three licensed classes of antiviral drugs for influenza virus infection: M2 channel inhibitors (e.g., amantadine), neuraminidase inhibitors (e.g., oseltamivir and zanamivir), and viral RNA polymerase inhibitors (e.g., favipiravir). But the emergence of drug-resistant viruses has become a serious problem. Adamantanes are no longer clinically used because most of viruses are resistant [6, 7]. Moreover, some seasonal viruses with high levels of resistance to oseltamivir and oseltamivir-resistant A(H7N9) viruses in humans have been identified [12]. Favipiravir is a broad-spectrum antiviral that was recently developed to prevent influenza virus infection, but it also still faces the issue of the emergence of resistant variants with its extensive use. Two mutations in the PB2 and PA genes that provide robust resistance to favipiravir have been identified in the laboratory setting [13]. Therefore, there is an urgent need to identify novel targets for influenza antiviral drug development.

Viruses rely on the host cellular machinery to complete their life cycles, and some host factors play pivotal roles in viral replication. Such host factors are ideal drug targets or therapeutic targets because they will not mutate under drug- or vaccine-mediated selective pressure. Screening and identifying key host factors required for viral replication will provide important insights into potential antiviral drug targets. Genome-wide screening methods have been widely used, including small interfering RNA (siRNA), proteomics, and insertional mutagenesis screening, to identify host factors involved in influenza virus infection and infections by other viruses such as HIV [14–20]. Since 2013, CRISPR/Cas9 technology has been gradually used to perform genome-wide screens for host factors of interest in mammalian cells [21, 22]. Unlike RNAi inhibition, CRISPR/Cas9 decreases gene expression at the DNA level, and can be used to study certain phenotypes that require knockout levels, or it can be used to study non-transcriptional regions and non-coding RNAs. CRISPR/Cas9 genome-wide screens have proven to be more efficient and reliable to identify host factors required for viral replication [23–28].

Here, we performed a genome-wide CRISPR/Cas9 screen in human lung epithelial cells (A549 cells) to identify host factors required for highly pathogenic H5N1 virus infection. We found that transmembrane protein immunoglobulin superfamily DCC subclass member 4 (IGDCC4) interacts with viral HA protein and facilitates viral internalization. H5N1 virus replication and virulence were impaired in IGDCC4-knockout mice.

## Results

### Establishment of Genome-wide CRISPR/Cas9 screening in A549 cells

To identify host factors required for influenza virus infection, we performed a genome-wide CRISPR/Cas9 screen in A549 cells as described previously [21]. An A549-Cas9 AAVS1 cell line stably expressing Cas9 protein was purchased from GeneCopoeia (Cat: SCL-76321-G2), and subcloned to obtain clones with high Cas9 protein expression based on copGFP intensity by using FACS sorting.

**Fig 1:**
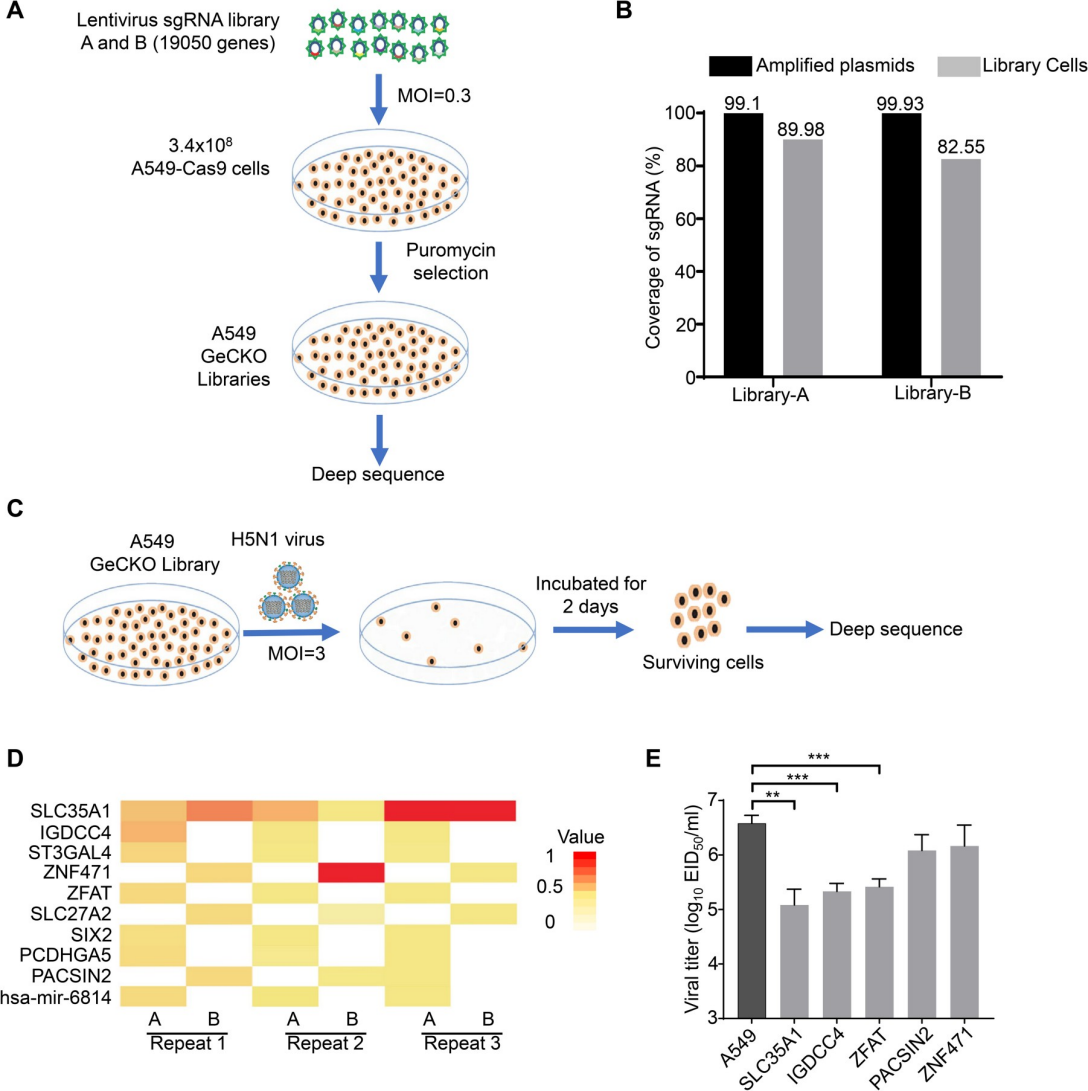
Genome-wide CRISPR/Cas9 screening of A549 cells for host factors required for highly pathogenic H5N1 influenza virus infection. (**A**) Schematic of the generation of the A549-GeCKO libraries by using lentivirus sgRNA libraries and A549 cells expressing Cas9 (A549-Cas9). A549-Cas9 cells were transduced with lentivirus containing sgRNA libraries A and B, respectively, and were maintained in puromycin for 14 days to generate the A549-GeCKO library, which was then deep sequenced. (**B**) Coverage of amplified library plasmids and GeCKO library cells compared with sgRNA lists from Addgene detected by high-throughput sequencing. (**C**) Schematic of screening host factors associated with H5N1 virus infection. (**D**) The heat map plots showing sgRNA abundance of the selected host factors in different screens. (**E**) H5N1 virus replication titers in different gene knockout cells. The cells were infected with H5N1 virus at an MOI of 0.01; the supernatants were collected at 48 h post-infection for viral titration in chicken embryos. The data shown are the means ± SDs of three biological repeats. The two-tailed unpaired *t*-test was used for the statistical analysis. **, *p* < 0.01, ***, *p* < 0.001.

We transduced the A549-Cas9 cells with lentivirus pools for human GeCKO v2 single guide RNA (sgRNA) libraries A and B, respectively. The two libraries contained, in total, 123,411 unique sgRNAs targeting 19,050 genes (www.addgene.org). To ensure the integrity of the cell libraries, each sgRNA covered at least 1,000 cells. Each cell library was cultured for 14 days with puromycin pressure to remove cells without transduced sgRNA. We then performed high-throughput sequencing for plasmid libraries with one amplification in *E*. *coli* and the transduced cell libraries to confirm library integrity (Fig 1A). The plasmid libraries A and B contained 99.10% and 99.93% sgRNAs, respectively, compared with the reference libraries from Addgene. Some sgRNAs may target host genes essential for cell survival, so the transduced cell libraries A and B were just maintained with 89.98% and 82.55% sgRNAs, respectively (Fig 1B). Thus, we generated pooled GeCKO libraries with sufficient coverage of sgRNAs in A549 cells (A549-GeCKO cell libraries) to be used to screen host factors essential for influenza virus infection.

### Screening and identifying host factors required for H5N1 influenza virus infection

Next, we infected the pooled A549-GeCKO cell libraries with A/Anhui/1/2005(H5N1) virus (H5N1 virus) at a multiplicity of infection (MOI) of 3; this virus was isolated from a patient with a fatal outcome in China in 2005 [29]. Surviving cells were reseeded and expanded for two more rounds of H5N1 virus infection. Then genomic DNA was extracted from the surviving cells, and integrated sgRNAs were amplified by PCR and sequenced (Fig 1C). We repeated the screening three times, and identified robust enrichment of 979 sgRNAs (≥15 reads) targeting 648 human genes (S1 Table), of which 10 host genes with the highest enrichment were identified in three independent experiments (Fig 1D). Two of these ten genes, SLC35A1 and ST3GAL4, have previously been identified by others [30, 31].

Host dependency factors acquired by using genome-scale CRISPR knockout screens must be further validated to exclude false positives due to bad sequencing readouts, copy number variants, or off-target sgRNAs. H5N1 virus replicated similarly in A549 cells transduced with lentivirus bearing the non-targeted gRNA (A549-NT) and wild-type A549 cells, indicating that lentivirus transduction does not affect influenza virus replication in A549 cells (S1 Fig). To further validate whether our candidate genes are important for influenza virus replication, we selected five of the top 10 candidates, including the previously reported SLC35A1 [30], and constructed their polyclonal knockout cell lines. The polyclonal cells for the five genes were infected with H5N1 virus at an MOI of 0.01. Supernatants were collected for virus titration at 48 h post-infection (p.i.). Viral titers in the SLC35A1-, IGDCC4-, and ZFAT-knockout cells were significantly lower than those in the control A549 cells, but knockout of PACSIN2 and ZNF471 did not significantly affect H5N1 virus replication (Fig 1E).

### IGDCC4 is important for the early step of influenza virus replication

**Fig 2:**
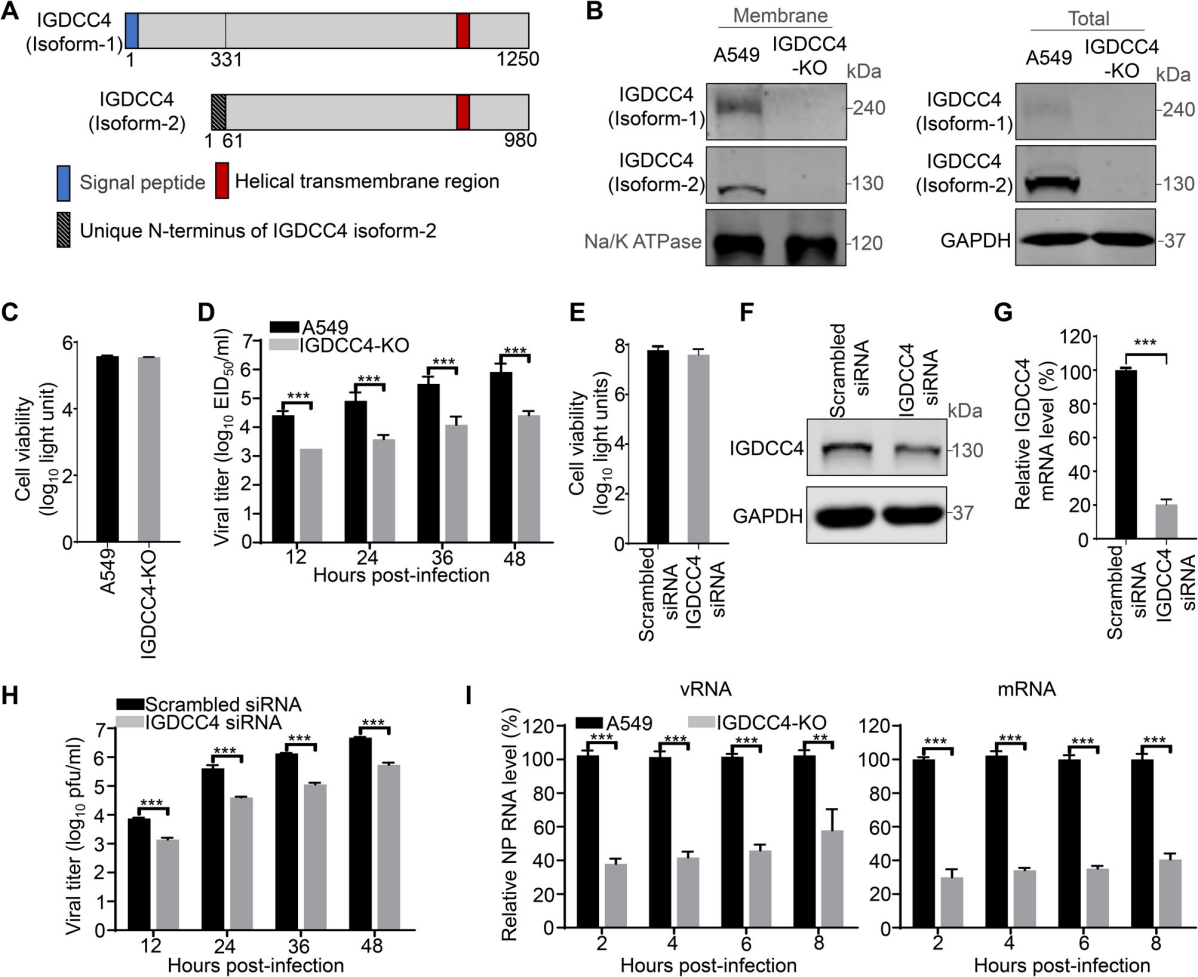
IGDCC4 is important for the early stage of H5N1 virus replication. (**A**) Schematic illustration of the proteins encoded by *IGDCC4*. The information on the proteins encoded by the *IGDCC4* gene was acquired from the UniProtKB website (accession number: Q8TDY8) (www.uniprot.org). The amino acids 62 to 980 in isoform 2 were identical to amino acids 332 to 1250 in isoform 1. (**B**) IGDCC4 expression level in A549 cells and IGDCC4-KO cells. Both the membrane proteins and the total proteins of the A549 cells and IGDCC4-KO cells were assessed by Western blotting to determine the IGDCC4 protein expression level. (**C**) The viability of IGDCC4-KO cells was measured by using the CellTiter-Glo assay and compared with that of the control A549 cells. (**D**) Replication of H5N1 virus in IGDCC4-KO and control A549 cells. IGDCC4-KO and A549 cells were infected with H5N1 virus at an MOI of 0.01; supernatants were collected at the indicated timepoints for virus titration in chicken embryos. (**E**) The viability of A549 cells transfected with the indicated siRNA was measured by using the CellTiter-Glo assay. (**F**) Knockdown of IGDCC4 by siRNA in A549 cells. The mRNA level of IGDCC4 in A549 cells transfected with siRNA targeting IGDCC4 was measured by qRT-PCR at 36 h post-transfection and was compared with that in A549 cells transfected with scrambled siRNA. (**G**) IGDCC4 expression level in A549 cells transfected with different siRNA. (**H**) Replication of H5N1 virus in IGDCC4 knockdown cells and A549 cells. A549 cells transfected with siRNA targeting IGDCC4 or scrambled siRNA were infected with H5N1 virus at an MOI of 0.01; supernatants were collected at the indicated timepoints for virus titration in MDCK cells by using plaque assays. (**I**). The effect of IGDCC4 on viral transcription and viral genome replication of H5N1 virus. IGDCC4-KO and A549 cells were infected with H5N1 virus at an MOI of 3. The mRNA and vRNA levels of the NP gene were detected by using qRT-PCR and were normalized with the value of A549 cells at the indicated timepoints. The data shown in panels B–I are the means ± SDs of three independent experiments or replicates. The two-tailed unpaired t-test was used for the statistical analysis. **, *p* < 0.01, ***, *p* < 0.001.

IGDCC4 may encode two proteins via alternative splicing (www.uniprot.org): isoform 1 is a single-pass type I transmembrane protein, and isoform 2 lacks a signal peptide and may be an intracellular protein. Compared with isoform 1, isoform 2 lacks 270 amino acids at the N-terminus, and its 61 amino acids at the N-terminus differ from amino acids 271–331 of isoform 1 (Fig 2A). The function of IGDCC4 in viral replication has never been studied. We therefore further investigated the effect of IGDCC4 on influenza virus infection. An IGDCC4 knockout A549 cell clone (IGDCC4-KO) was generated by using CRISPR/Cas9 and knockout of both IGDCC4 isoform 1 and isoform 2 was confirmed by Western blotting with a mouse anti-IGDCC4 antibody (Fig 2B). IGDCC4 knockout had no effect on cell viability as measured by using a luminescent cell viability assay (Fig 2C).

To investigate the effect of IGDCC4 on influenza virus replication, IGDCC4-KO and A549 cells were infected with H5N1 virus at an MOI of 0.01. The supernatants were collected for viral titration at different timepoints after infection. The titers of H5N1 virus in IGDCC4-KO cells were 14.7-fold, 21.5-fold, 26.1-fold, and 31.6-fold lower than those in the A549 cells at 12, 24, 36, and 48 h p.i., respectively (Fig 2D). To further confirm these results, we synthesized and tested an IGDCC4 siRNA (Fig 2E–2G), and found that the replication of H5N1 virus in the IGDCC4 siRNA-treated cells was 5.4-fold, 10.3-fold, 11.8-fold, and 8.8-fold lower than that of the virus in the A549 cells at 12, 24, 36, and 48 h p.i., respectively (Fig 2H). These results demonstrate that the cellular protein IGDCC4 promotes influenza virus replication.

To determine which stage of the influenza virus life cycle is affected by IGDCC4, IGDCC4-KO and control A549 cells were infected with H5N1 virus at an MOI of 3, and the levels of vRNA and mRNA of the viral NP gene in the cells were measured at 2, 4, 6, and 8 h p.i. by using quantitative reverse transcription PCR (qRT-PCR). At these four timepoints, the levels of both types of viral RNA in IGDCC4-KO cells were significantly lower than those in A549 cells (Fig 2I). At 2 h p.i., the early stage of the viral life cycle, the vRNA in the cells was mainly derived from infecting virus rather than progeny virus, and the lower level of vRNA in IGDCC4-KO cells at this timepoint indicates that the host factor IGDCC4 was most likely involved in the early stage of the viral life cycle.

### Knockout of IGDCC4 reduces the nuclear targeting of NP

**Fig 3:**
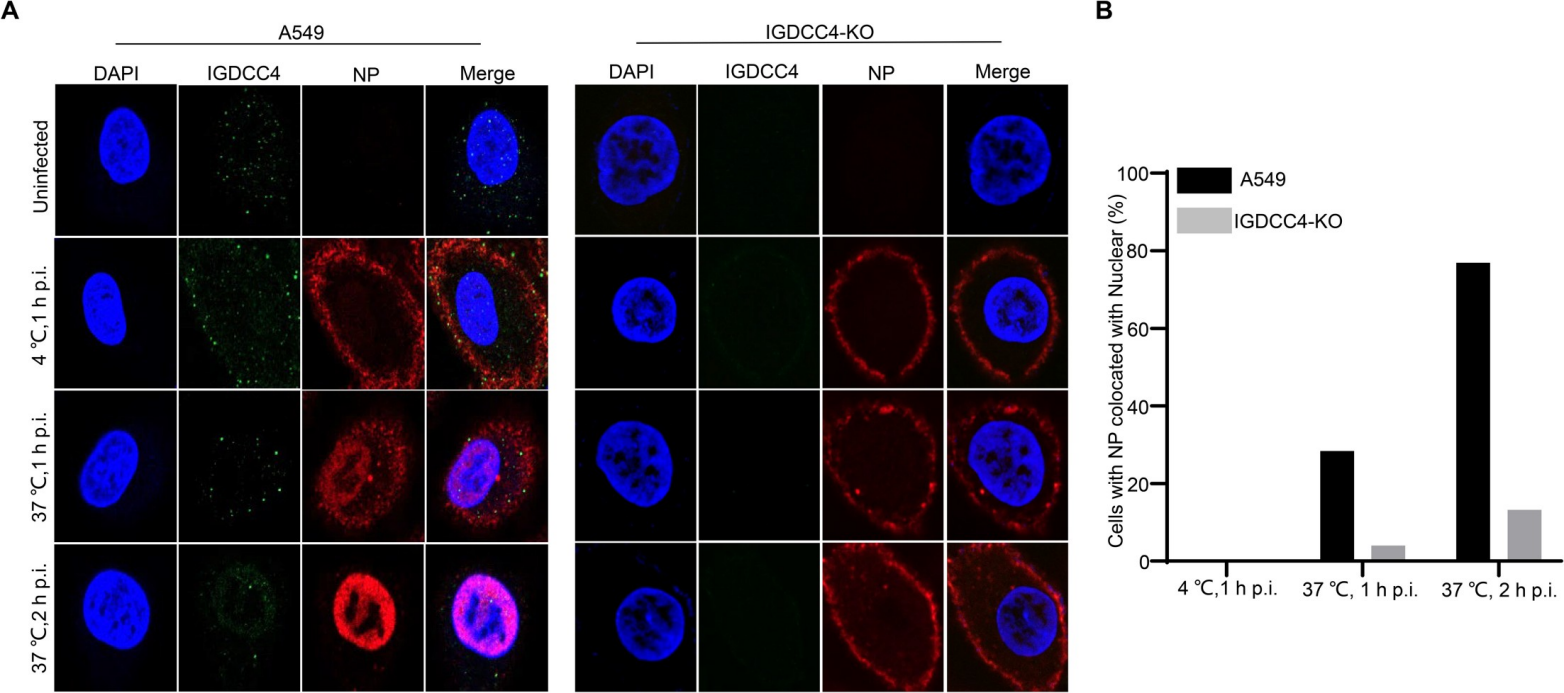
Colocalization of IGDCC4 and viral NP protein in A549 cells and IGDCC4-KO cells infected with H5N1 virus at different timepoints. **(A)** Cells were infected with H5N1 virus at an MOI of 5 and incubated at the indicated temperature and for the indicated time as described in the text. They were then fixed and stained with a rabbit anti-NP antibody and a mouse anti-IGDCC4 antibody, followed by incubation with Alexa Fluor 633 goat anti-rabbit IgG(H+L) (red) and 488 donkey anti-mouse IgG(H+L) (green). The nuclei were stained with DAPI (blue). (**B**) Quantitative analysis of NP localization in virus-infected cells. The ratio of cells showing colocalization of virus NP and nucleus was calculated from 100 virus-infected cells.

We investigated the cellular distribution of IGDCC4 and viral NP protein at the early stage of virus infection by using confocal fluorescent microscopy. A549 cells and IGDCC4-KO cells infected with the H5N1 virus at an MOI of 5 were incubated at 4°C for 1 h and then at 37°C for 2 h. The cells were fixed at different incubation timepoints and stained with specific antibodies to observe the localization of the IGDCC4 and viral NP proteins. IGDCC4 was detected in the uninfected and infected A549 cells at 1 h p.i., it was detected at a reduced level at 2 h p.i. (1 h post-incubation at 37°C), and was nearly undetectable at 3 h p.i. (2 h post-incubation at 37°C) (Fig 3A). In contrast, IGDCC4 was not detected in IGDCC4-KO cells at any timepoint (Fig 3A). Viral NP was clearly visible in the A549 cells at 1 h p.i., and accumulated in the nucleus of 28% and 77% of the A549 cells at 2 h (1 h post-incubation at 37°C) and 3 h p.i. (2 h post-incubation at 37°C), respectively (Fig 3B). In contrast, viral NP was only detected in the nucleus of 4% and 13% of IGDCC4-KO cells at 2 h (1 h post-incubation at 37°C) and 3 h p.i. (2 h post-incubation at 37°C), respectively (Fig 3B). These results further demonstrate that IGDCC4 is involved in the early stage of the influenza virus life cycle.

### IGDCC4 promotes influenza virus internalization

**Fig 4:**
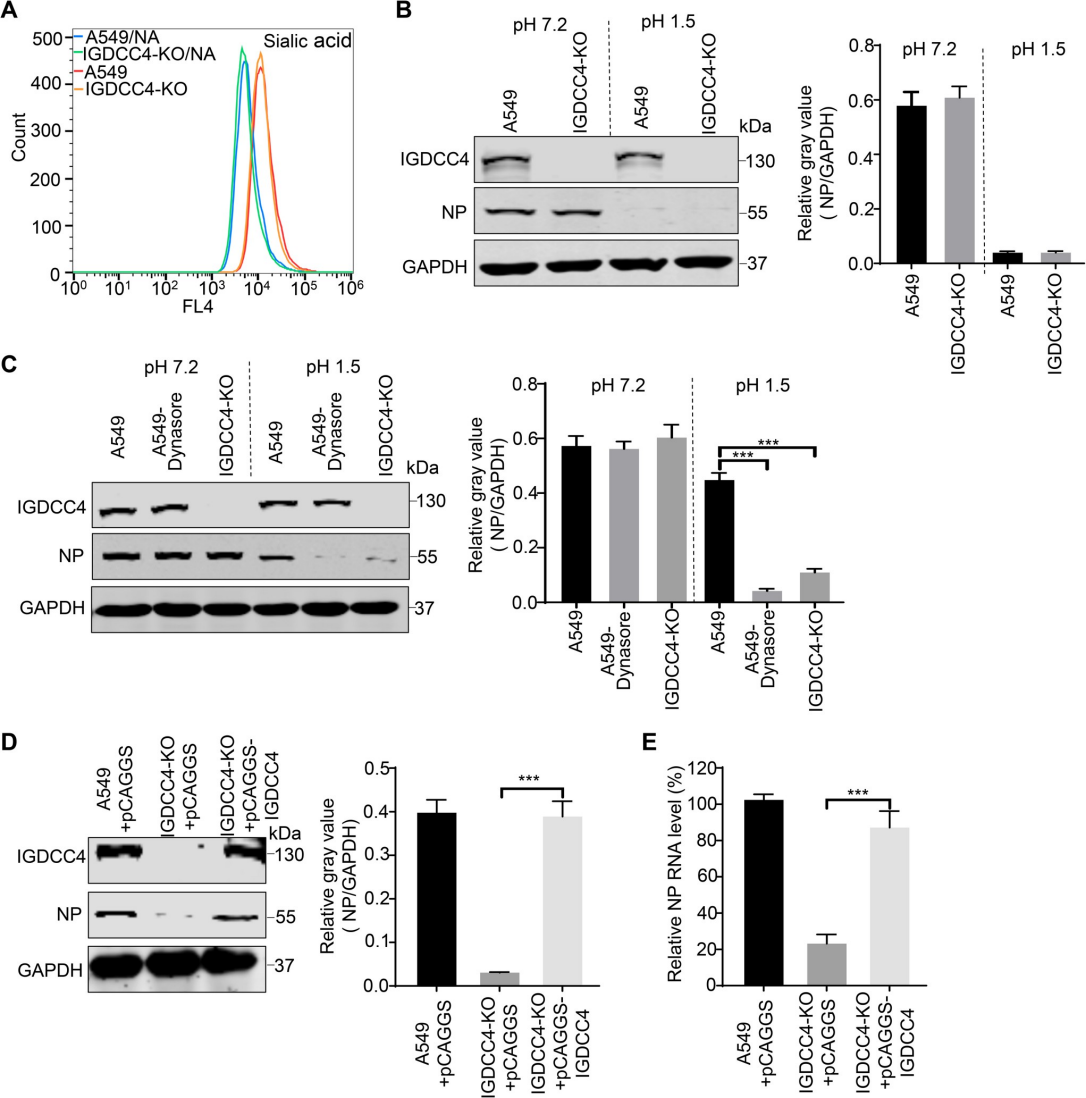
IGDCC4 is required for influenza virus internalization. (**A**) Sialic acid expression in different cells. IGDCC4-KO and A549 control cells treated with or without neuraminidase (NA) were stained with Alexa Fluor 647 conjugated with wheat germ agglutinin, and after three washes with PBS, the cells were suspended to detect WGA binding by use of flow cytometry. (**B**) NP protein of viruses attached to the surface of IGDCC4-KO and A549 cells. IGDCC4-KO and A549 cells were incubated with H5N1 virus at an MOI of 5 at 4°C for 1 h and then washed with cold PBS (pH = 7.2) or cold acidic PBS (pH = 1.5), which can elute virus particles on the cell surface that have not been internalized. The cells were collected and the NP protein of the viruses attached to the cells was evaluated by Western blotting. (**C**) NP protein of viruses internalized into IGDCC4-KO and A549 cells. A549 cells treated with or without dynasore and IGDCC4-KO cells were incubated with H5N1 virus at an MOI of 5 at 4°C for 1 h and at 37°C for 1 h. After being washed with cold PBS (pH = 7.2) or acidic PBS (pH = 1.5), the cells were collected and the NP protein level of the total attached and internalized viruses (normal PBS washed) or the internalized viruses (acidic PBS washed) was determined by using Western blotting. (**D**) Overexpression of IGDCC4 restores the internalization of influenza virus into IGDCC4-KO cells. A549 cells and IGDCC4-KO cells transfected with the indicated plasmids were infected with H5N1 virus at an MOI of 5. After being incubated at 4°C for 1 h and 37°C for 1 h, the cells were washed three times with ice-cold acidic PBS. The washed cells were then collected to detect the NP protein level by Western blotting, and (**E**) the RNA level by qRT-PCR. The band intensities of the Western blots from three assays were quantified by using ImageJ software, and the relative gray values of NP to GAPDH are presented. The data shown represent or are from three independent experiments or replicates (means ± SDs). The two-tailed unpaired t-test was used for the statistical analysis. ***, *p* < 0.001.

Binding of HA protein to the sialic acid receptors on the cell surface is the first step for influenza virus to invade the host cell [32]. Because wheat germ agglutinin (WGA) specifically recognizes sialic acid moieties [33], we used Alexa Fluor 647-conjugated WGA and flow cytometry to examine the expression of sialic acid receptor on the surface of IGDCC4-KO and A549 cells treated with or without neuraminidase (NA). We found that the expression level of sialic acid receptors on the surface of NA-treated cells was clearly lower than that on the untreated cells, but the level of sialic acid receptors on the surface of IGDCC4-KO and A549 cells was comparable (Fig 4A).

To test whether knockout of IGDCC4 affects the attachment of influenza virus to the cell membrane, IGDCC4-KO and A549 cells were incubated with H5N1 virus at an MOI of 5 at 4°C for 1 h and then washed with cold PBS (pH = 7.2) or cold acidic PBS (pH = 1.5), which can elute virus particles on the cell surface that have not been internalized [14]. The cells were collected and the NP protein of viruses attached to the cells was evaluated by Western blotting. As shown in Fig 4B, in the PBS (pH = 7.2) washed cells, the level of NP protein attached to the surface of IGDCC4-KO cells was comparable to that of A549 cells, but in the cold acidic PBS washed cells, the level of NP protein of both cells was nearly not detectable, indicating that IGDCC4 is not needed for H5N1 virus to attach the cell surface.

To examine the effect of IGDCC4 on virus internalization, A549 cells treated with or without dynasore, a specific endocytosis inhibitor, and IGDCC4-KO cells were incubated with H5N1 virus at an MOI of 5 at 4°C for 1 h and at 37°C for 1 h. After being washed with cold PBS (pH = 7.2) or acidic PBS (pH = 1.5), the cells were collected and the NP protein level of the total attached and internalized viruses (normal PBS washed) or the internalized viruses (acidic PBS washed) was determined by using Western blotting. In the PBS (pH = 7.2) washed cells, the NP level was comparable; in the acidic PBS washed cells, the NP level in IGDCC4-KO and dynasore-treated A549 cells was comparable, but clearly lower than that in control A549 cells (Fig 4C), indicating that most of the influenza virus particles were not internalized into the IGDCC4-KO or dynasore-treated A549 cells.

We further investigated whether overexpression of IGDCC4 could restore the endocytosis of influenza virus in IGDCC4-KO cells. We found that the NP protein level (Fig 4D) and RNA level (Fig 4E) of the viruses internalized in the pCAGGS-IGDCC4 transfected IGDCC4-KO cells were comparable to those in A549 cells transfected with pCAGGS, but significantly higher than those in IGDCC4-KO cells transfected with pCAGGS. These results demonstrate that IGDCC4 indeed promotes the internalization of influenza virus.

### IGDCC4 interacts with the HA protein of influenza virus

**Fig 5:**
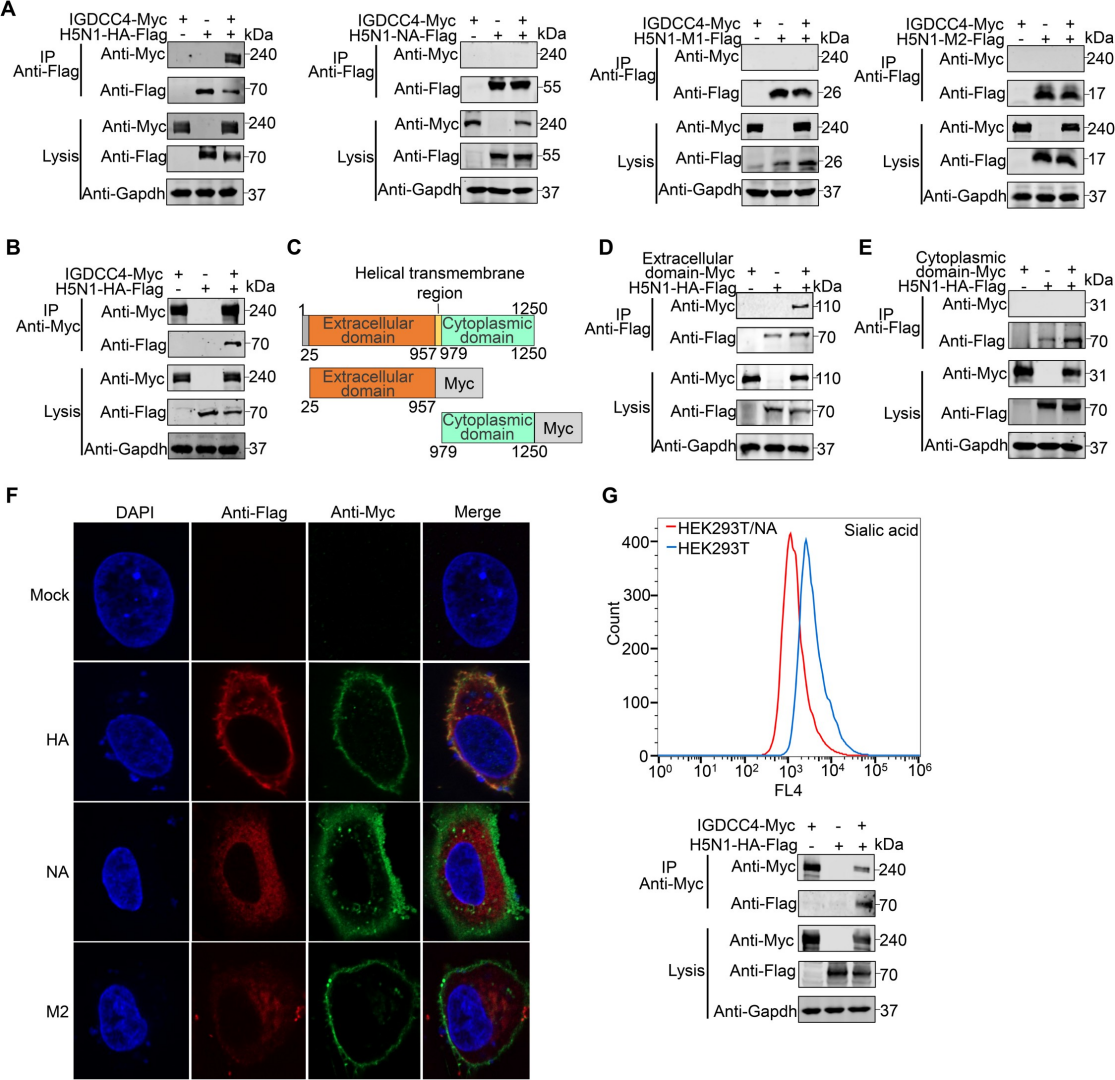
IGDCC4 interacts with the HA protein of influenza virus. (**A**) Interaction of IGDCC4 with the HA, NA, M1, and M2 proteins of H5N1 virus. HEK293T cells were transfected individually or in combination with plasmids for the expression of IGDCC4 fused with Myc tag and viral proteins fused with Flag tag. (**B**) IGDCC4 interaction with the HA protein of H5N1 virus was confirmed by immunoprecipitation with the anti-Myc antibody. (**C**) Schematic presentation of IGDCC4. (**D**) The extracellular domain of IGDCC4 interacts with the HA of H5N1 virus. (**E**) The endocytic domain of IGDCC4 does not interact with the HA of H5N1 virus. (**F**) A549 cells were co-transfected with a plasmid expressing IGDCC4 and a plasmid expressing HA, NA, or M2 of H5N1 virus, and the interaction of IGDCC4 with these proteins was confirmed by using a colocalization assay. (**G**) The HA of H5N1 virus interacted with IGDCC4 expressed in HEK293T cells treated with neuraminidase. The data shown are representative of three independent experiments.

Given that IGDCC4 is required for influenza virus internalization, it may interact with one of the surface proteins of the influenza virus. We therefore investigated whether IGDCC4 interacts with the HA, NA, or M protein of H5N1 virus by using a co-immunoprecipitation assay. HEK293T cells were transfected with plasmids bearing Flag-tagged HA, NA, M1, or M2 of H5N1 virus individually or together with a plasmid bearing Myc-tagged IGDCC4. Cell lysates were immunoprecipitated with an anti-Flag mAb, and then Western blotted with a rabbit polyclonal antibody against Flag or Myc tag. We found that Myc-tagged IGDCC4 was co-immunoprecipitated with Flag-tagged HA when they were co-expressed, but was not co-immunoprecipitated with Flag-tagged NA, M1, or M2 (Fig 5A). The interaction between IGDCC4 and HA was further confirmed by performing a reverse co-immunoprecipitation experiment with an anti-Myc monoclonal antibody (Fig 5B). Of note, although both IGDCC4 isoform 1 and isoform 2 were clearly detected in the IGDCC4-overexpressing cells (S2 Fig), only isoform 1 was co-immunoprecipitated with H5N1 HA.

IGDCC4 isoform 1 contains 1,250 amino acids: its signal peptide is located at amino acids 1–24, the extracellular domain is located at amino acids 25–957, the transmembrane domain is located at amino acids 958–978, and the cytoplasmic domain is located at amino acids 979–1250 (Fig 5C) (https://www.uniprot.org). To identify which domain of IGDCC4 interacts with the HA protein of H5N1 virus, we constructed two plasmids respectively bearing the IGDCC4 extracellular domain and the IGDCC4 cytoplasmic domain fused with the C-terminal of Myc (Fig 5C). Co-immunoprecipitation revealed that the IGDCC4 extracellular domain binds strongly with the HA protein (Fig 5D), whereas the cytoplasmic domain does not bind to the HA protein (Fig 5E).

The interaction of IGDCC4 and HA was further confirmed by using a colocalization assay. When A549 cells were co-transfected with a plasmid expressing IGDCC4 and a plasmid expressing HA, NA, or M2 of H5N1 virus, HA and IGDCC4 clearly colocalized at the cell membrane; however, colocalization of IGDCC4 with NA or M2 was not observed (Fig 5F). The co-immunoprecipitation of IGDCC4 and H5N1 virus HA expressed in HEK293T cells treated with neuraminidase indicated that the HA interaction with IGDCC4 is not affected by sialic acid binding (Fig 5G).

**Fig 6:**
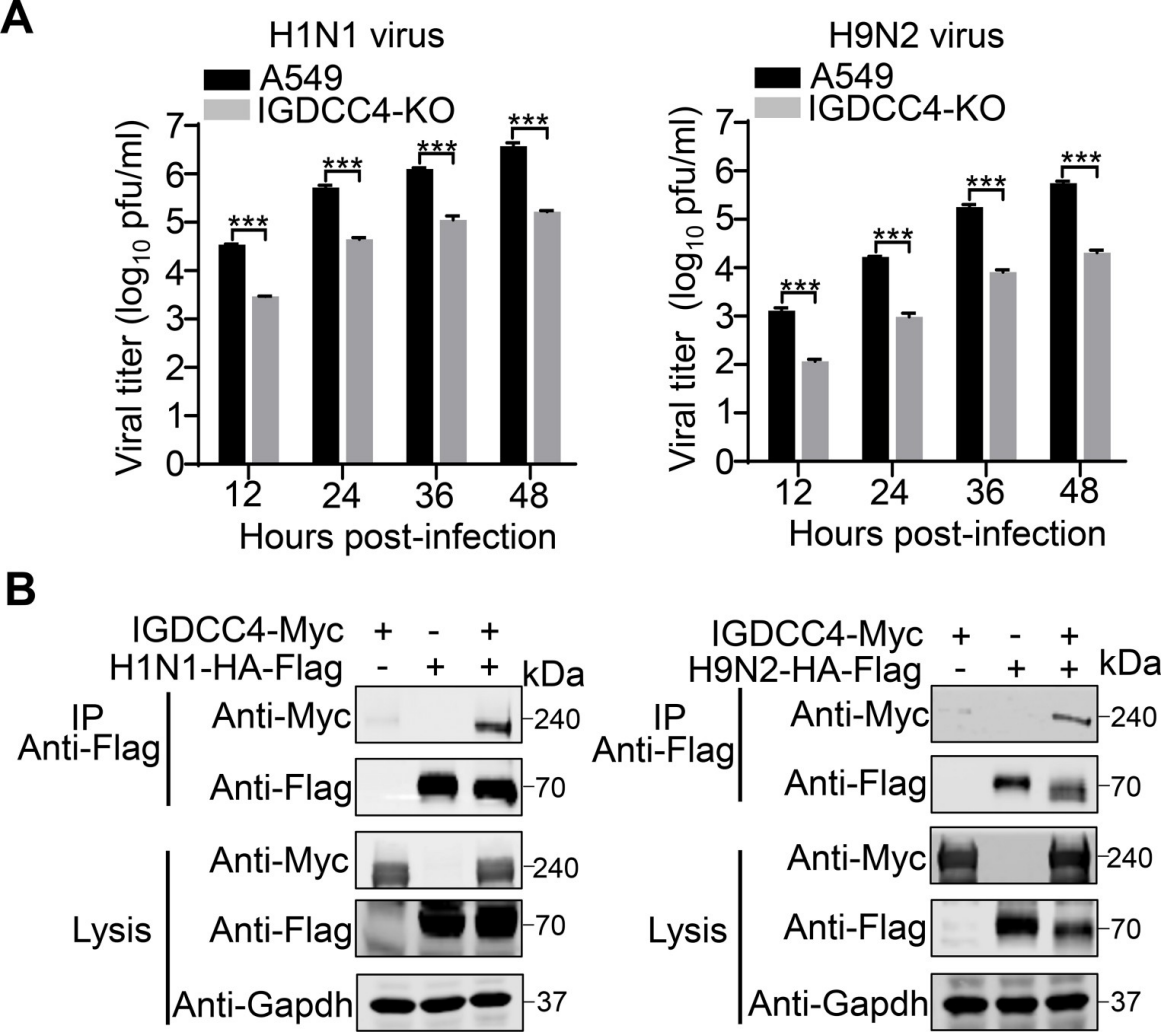
Effect of IGDCC4 on H1N1 influenza virus and H9N2 influenza virus. (**A**) Replication titers of H1N1 virus and H9N2 virus in A549 cells and IGDCC4-KO cells. A549 cells and IGDCC4-KO cells were infected with the indicated virus at an MOI of 0.01 and the supernatants were harvested at the indicated time for virus titration in MDCK cells. The data shown are from three biological replicates (means ± SDs) (**B**) Interaction of IGDCC4 with the HA protein of H1N1 virus and H9N2 virus. HEK293T cells were transfected individually or in combination with plasmids for the expression of IGDCC4 fused with Myc tag and viral HA protein fused with Flag tag. Cell lysates were immunoprecipitated with a mouse anti-Flag monoclonal antibody. The data shown are representative of three independent experiments. The two-tailed unpaired *t*-test was used for the statistical analysis. ***, *p* < 0.001.

The above studies indicated that IGDCC4 affects the replication of the H5N1 virus by interacting with its HA and promoting its internalization. Does IGDCC4 also affect the replication of influenza viruses of other subtypes? To answer this question, we compared the replication of an H1N1 virus (A/WSN/1933) and an H9N2 virus (A/chicken/Jiangsu/C4258/2012) in control A549 cells and IGDCC4-KO cells, and found that the viral titers of H1N1 virus and H9N2 virus in the A549 cells were significantly higher than those in the IGDCC4-KO cells (Fig 6A and 6B). We further found that IGDCC4 interacts with H1 HA and H9 HA in co-immunoprecipitation experiments (Fig 6C and 6D). These results suggest that IGDCC4 may interact with the HA of different subtypes of influenza viruses and facilitate their internalization.

### IGDCC4 does not affect the internalization of vesicular stomatitis virus (VSV) and transferrin

**Fig 7:**
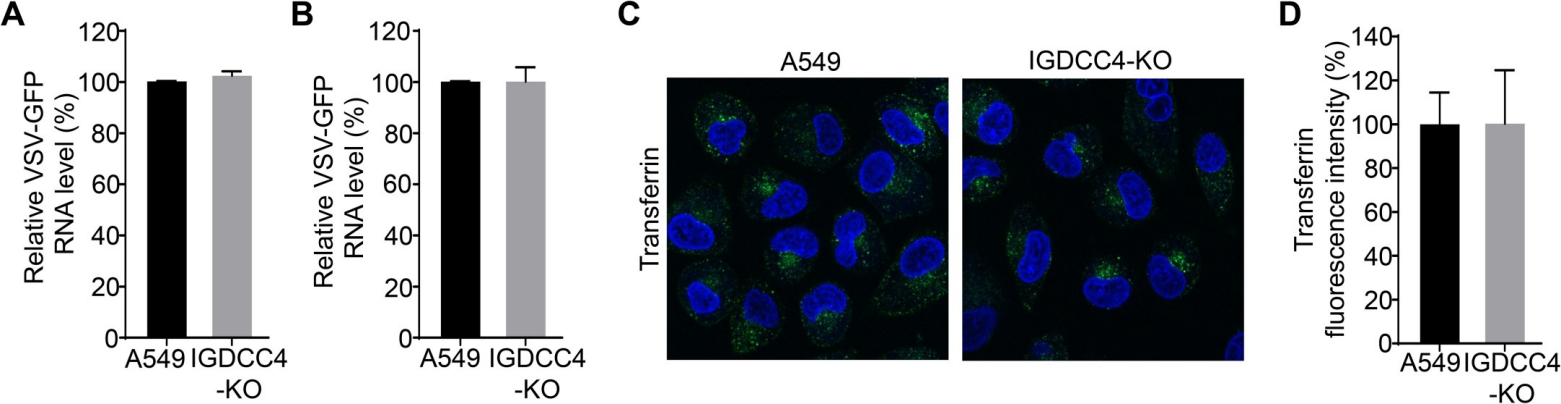
IGDCC4 does not affect the internalization of vesicular stomatitis virus-green fluorescent protein (VSV-GFP) and transferrin. The A549 cells and IGDCC4-KO cells infected with VSV-GFP at an MOI of 10 were incubated and washed differently before being collected for detecting viral attachment (**A**) or (**B**) internalization by using qRT-PCR, the values of IGDCC4-KO cells were normalized with that of A549 cells. Data shown are from three replicates (means ± SDs). (**C**) and (**D**) Internalization of transferrin in IGDCC4-KO and A549 cells were evaluated by incubating cells with 50 μg/ml fluorescently labeled transferrin at 37°C for 30 min before receiving an acid wash to quench extracellular transferrin and were subsequently processed for confocal microscopy. Automatic image analysis quantified a total of 100 cells in three independent experiments and normalized to the A549 cells. The data shown in A, B, and D are from three independent experiments or biological replicates (means ± SDs).

A549 cells and IGDCC4-KO cells infected with VSV-green fluorescent protein (VSV-GFP) at an MOI of 10 were inoculated for 1 h at 4°C or for 1 h at 4°C and then 1h at 37°C for evaluating the attached viruses or internalized viruses. We found that the RNA level of VSV-GFP virus attached to IGDCC4-KO and A549 cells was comparable (Fig 7A), and that the RNA level of VSV-GFP virus internalized to IGDCC4-KO cells and A549 cells was also comparable (Fig 7B). To further investigate whether IGDCC4 is important for endocytosis of cargo in general, we compared the internalization of transferrin in A549 and IGDCC4-KO cells, and we found that there was no difference in the internalization of transferrin between the A549 cells and the IGDCC4-KO cells (Fig 7C and 7D). These results suggest that IGDCC4-mediated endocytosis may be cargo-specific.

### IGDCC4-knockout mice show increased resistance to H5N1 virus infection

**Fig 8:**
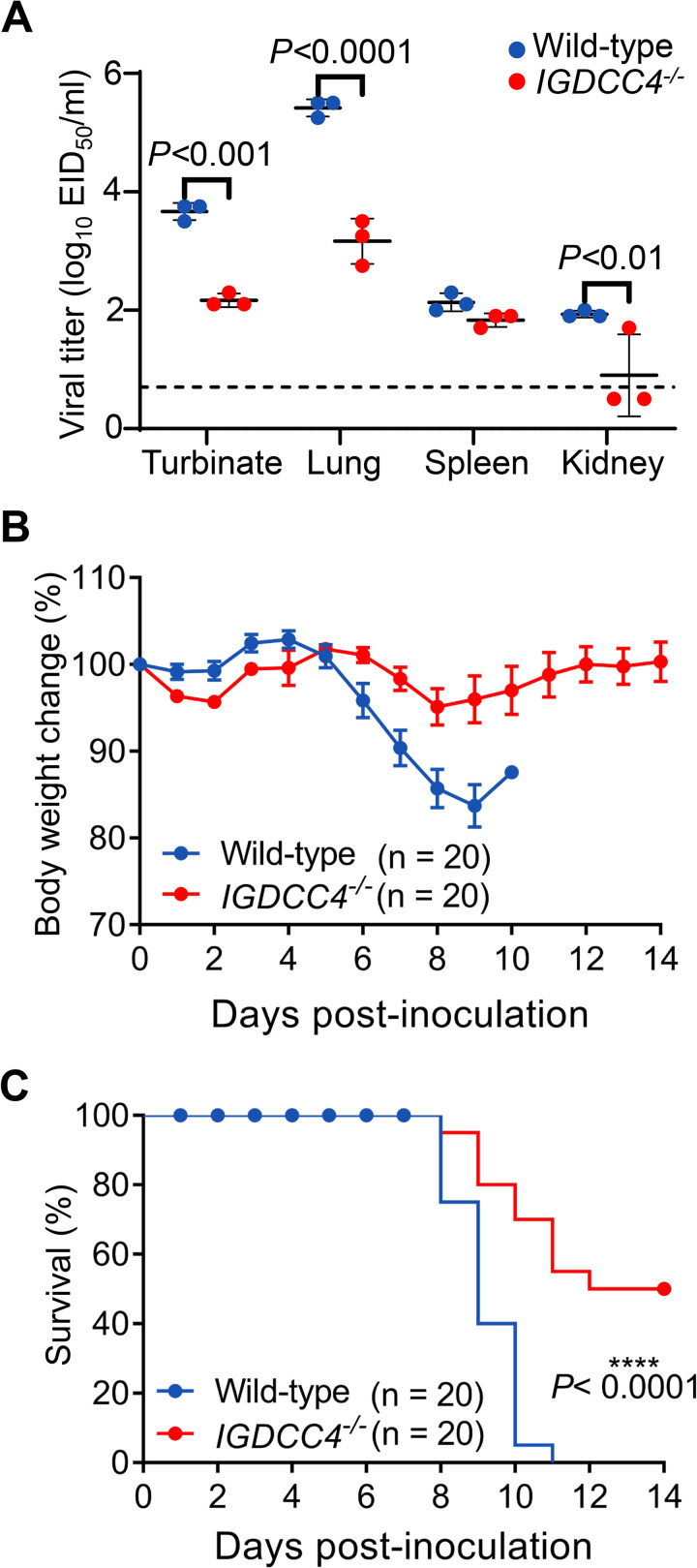
Replication and lethality of H5N1 virus in wild-type mice and IGDCC4 knockout mice. (**A**) Viral titers in organs of different mice. Wild-type mice and IGDCC4 knockout mice were euthanized on Day 3 post-infection with 5 MLD_50_ of H5N1 virus, and their organs were collected for virus titration in chicken embryos. The data are presented as means ± SDs for organ samples of three mice. (**B**) Body weight change of wild-type mice and IGDCC4 knockout mice after H5N1 virus infection. The values are means ± SDs from surviving mice at the indicated timepoints. (**C**) Lethality of H5N1 virus to wild-type mice and IGDCC4 knockout mice. The two-tailed unpaired *t*-test was used for the statistical analysis.

Our *in vitro* studies demonstrated that IGDCC4 plays an important role in the endocytosis of H5N1 virus infection; however, does IGDCC4 affect viral replication *in vivo*? To answer this question, we generated IGDCC4-knockout (*IGDCC4*^−/−^) mice on the C57BL/6J background and evaluated H5N1 virus replication and lethality in both wild-type and *IGDCC4*^−/−^ mice. Virus replication was detected in the nasal turbinates, lungs, and spleen of all wild-type and *IGDCC4*^−/−^ mice tested, but the titers in the nasal turbinates and lungs of the *IGDCC4*^−/−^ mice were significantly lower than those in the wild-type mice (Fig 8A). H5N1 virus was isolated in the kidneys of all three wild-type mice, whereas it was only detected in the kidneys of one of the three *IGDCC4*^−/−^ mice (Fig 8A). All 10 wild-type mice started to loss body weight from day 6 p.i. and died between days 8 and 11 p.i., whereas the *IGDCC4*^−/−^ mice lost less than 10% of their body weight; five died between days 8 and 12 p.i. but the other five mice recovered and survived. We repeated the lethality study with groups of 10 mice and found that the results were reproducible; the combined data of the two experiments are shown in Fig 8B and 8C. Our results show that IGDCC4 facilitates H5N1 virus infection *in vivo* and that *IGDCC4*^−/−^ mice exhibit increased resistance to H5N1 virus infection.

## Discussion

In this study, we used genome-wide CRISPR/Cas9 knockout screening technology to explore the host proteins that are involved in lethal H5N1 influenza virus infection. By using this approach, we identified a novel host factor (IGDCC4) that facilitates the endocytosis of influenza virus infection. IGDCC4 interacted with the viral HA protein and significantly affected the internalization of influenza virus into cells. Importantly, we further showed that IGDCC4 facilitates H5N1 virus infection *in vivo* and that *IGDCC4*^−/−^ mice have increased resistance to H5N1 virus infection. Our study suggests that IGDCC4 is an important host factor that promotes influenza virus endocytosis.

Different techniques, including genome-wide siRNA screens [34–41], haploid cell genetic screens [42], short hairpin RNA screens [15, 43], and the newly developed CRISPR/Cas9 screens [30, 44, 45], have been used to identify the host factors involved in influenza virus replication. Due to the differences in cell type, virus strain, library type and diversity, timing of analysis, readout report analysis, screening thresholds, and statistical analyses, the results of different screening studies vary greatly [34, 46]. Both our study and the study by Han et al. [30]involved GeCKO screening in A549 cells to determine host factors required for H5N1 virus infection, but we used a highly pathogenic H5N1 virus, whereas Han et al. used a low pathogenic H5N1 virus. Among the top ten candidates identified in our study, only SLC35A1 and ZNF471 were also identified in Han’s study [30]. It is not clear whether these differences are entirely due to the different viral strains used in these two studies.

IGDCC4 is extensively expressed in many different cell types (https://bgee.org/), and our study clearly indicates that IGDCC4 promotes the internalization of influenza viruses. Since the influenza virus life cycle involves various steps, including attachment, internalization, uncoating, transcription and translation of the viral genome, assembly, and budding [34], numerous different host factors contribute to these steps and affect the susceptibility of cells to influenza virus. Therefore, endogenous expression of IGDCC4 in cell types may be positively correlated with the susceptibility of cells to influenza virus infection, but exogenous expression of IGDCC4 may not increase the susceptibility of cells to influenza virus infection.

Influenza virus infection is initiated by the attachment of the viral HA protein to the sialic acid receptor on the host cell surface; however, previous studies have indicated that the binding of influenza virus to sialylated cell surface proteins does not always result in receptor-mediated internalization [47, 48], suggesting that the engagement of specific cellular signaling is required to initiate virus endocytosis. In this study, we found that IGDCC4 interacts with the HA of influenza virus and mediates the endocytosis of influenza virus; however, this interaction does not play a role in the initial attachment of influenza virus to the cell surface, because the virus attached to wild-type A549 cells similarly to IGDCC4-KO cells. Our study thus suggests that influenza virus attachment and endocytosis may be mediated by different cellular proteins.

Influenza virus exploits multiple endocytic pathways for infection, but most of the virus particles enter cells through clathrin-mediated endocytosis [49]. It has been unclear how the signal of viral binding is transmitted across the plasma membrane to initiate the recruitment of clathrin and associated factors and induce the formation of clathrin-coated pits at the viral binding site. Previous studies have suggested that transmembrane proteins bearing endocytic signals in their cytosolic domains could be recognized by adaptors of clathrin located in the inner layer of clathrin coats and thereby could be internalized through clathrin-mediated endocytosis [50]. We found that there are three endocytic signals in the cytosolic domain of IGDCC4 that could be recognized by the adaptor protein 2 (AP2) of clathrin: two “tyrosine-based” YXXØ signals (1100YDAI1103 and 1210YPDL1213) and one [DE]XXXL[LI] signal (1162EAPDLI1167) (In this notation, amino acids are represented in the single-letter code, X indicates any amino acid, Ø indicates an amino acid with a bulky hydrophobic side chain, and the brackets mean that either amino acid is allowed at that position) (S3A Fig). This finding implies that the internalization of IGDCC4 may be clathrin-mediated. However, elimination of the three endocytic signals in the cytosolic domain of IGDCC4 did not affect the internalization of influenza virus (S3B–S3E Fig), suggesting that direct recognition of these endocytic signals by AP2 may not be the mechanism for IGDCC4-mediated internalization. Wang et al. previously reported that the membrane protein free fatty acid receptor 2 (FFAR2) plays a role in clathrin-mediated influenza virus endocytosis; however, FFAR2 does not directly associate with the AP2 adaptor complex, but relies on β-arrestin1 to link with the AP2-B1 subunit of the AP2 complex [14]. It is possible that other host factors may also be involved in the IGDCC4-mediated endocytosis of influenza virus, which deserves further study.

In addition to IGDCC4 and FFAR2, the voltage-dependent Ca2+ channel Cav1.2 was also reported to bind to the HA protein of H1N1 virus A/Puerto Rico/8/34 (PR8) and to mediate PR8 virus entry into mammalian cells. Animal studies have shown that intranasal administration of the Cav1.2 inhibitor diltiazem (20 mg/kg) is effective as prophylaxis against PR8 virus infection, because diltiazem treatment reduced virus replication by 4-fold in nasal lavage fluid and prolonged survival, even though the mice still lost nearly 30% of their body weight and 5 of 6 mice ultimately died during the 14-day observation [51]. The IGDCC4 knockout mice have increased resistance to H5N1 virus, with 50% of mice surviving a lethal challenge, indicating IGDCC4 plays a key role in mediating influenza virus infection.

In summary, we have identified a novel transmembrane protein, IGDCC4, that plays an important role in mediating influenza virus infection. This finding will be of value to the development of novel antiviral therapies.

## Materials and methods

### Ethics statement

All experiments with H5N1 virus were conducted in the enhanced animal biosafety level 3 (ABSL3) facility in the Harbin Veterinary Research Institute (HVRI) of the Chinese Academy of Agricultural Sciences (CAAS), which is approved for such use by the Ministry of Agriculture and Rural Affairs of China and the China National Accreditation Service for Conformity Assessment. The protocol for the animal studies was approved by the Committee on the Ethics of Animal Experiments of the HVRI, CAAS.

### Cells and viruses

Human embryonic kidney cells (HEK293T cells), human lung carcinoma cells (A549 cells), and Madin-Darby canine kidney cells (MDCK cells) were cultured in DMEM (Gibco, Cat.no. C11995500BT) supplemented with 10% fetal bovine serum (FBS; Gibco, Cat. no. 10099141) and 1% penicillin/streptomycin (P/S; Gibco, Cat.no. 15140122). The A549-Cas9 AAVS1 cell line was purchased from GeneCopoeia (Cat.no. SCL-76321-G2). Isolation of A/Anhui/1/2005 (H5N1) (H5N1 virus) and A/chicken/Jiangsu/C4258/2012(H9N2) (H9N2 virus) has been reported previously [52, 53], A/WSN/33(H1N1) (H1N1 virus) was kindly provided by Dr. Yoshihiro Kawaoka. GFP-expressing vesicular stomatitis virus (VSV-GFP) virus was generated in our laboratory as reported previously [54].

### Primers

The sequences of the primers used in this study are listed in Table 1.

**Table 1:** Sequence information of primers used in this study^a^.

Purpose	Primer sequence	
	Forward (5’ to 3’)	Reverse (5’ to 3’)
SLC35A1 knockout	CACCG***ATAGCGCCAAACCCTAATAA***	AAAC***TTATTAGGGTTTGGCGCTAT***C
IGDCC4 knockout	CACCG***CGTACTCGATACACCTTGTT***	AAAC***AACAAGGTGTATCGAGTACG***C
	CACCG***CAGTGGCGAAGTCGCGCGTG***	AAAC***CACGCGCGACTTCGCCACTG***C
ZFAT knockout	CACCG***GAGAGGAGTTCCGACTGATT***	AAAC***AATCAGTCGGAACTCCTCTC***C
PACSIN2 knockout	CACCG***AGAAACGCCTTCGCTTCTTC***	AAAC***GAAGAAGCGAAGGCGTTTCT***C
ZNF471 knockout	CACCG***TCATAAAGAAACCATCACTA***	AAAC***TAGTGATGGTTTCTTTATGA***C
Non-targeting control	CACCG***AAGCGGGCACACATGACAAG***	AAAC***CTTGTCATGTGTGCCCGCTT***C
IGDCC4 siRNA knockdown	GUCAUUGAAGGCAACUAUU	AAUAGUUGCCUUCAAUGAC
NP vRNA amplification	ACCCTTGCATCAGAGAGCAC	TTGGCGTCAAGCGAACAATG
NP mRNA amplification	AGCGAACAATGGAGAGGACG	TTGGGAGAGTTGACCCTTGC
IGDCC4 mRNA amplification	ATCCCCCTGCCCTCTAGGA	CTAGGCAGAGGAGGAGACCG
GAPDH mRNA amplification	GGAGCGAGATCCCTCCAAAAT	GGCTGTTGTCATACTTCTCATGG
VSV RNA amplification	GTGACGGACGAATGTCTCATAA	TTTGACTCTCGCCTGATTGTAC
IGDCC4 detection in mouse	GCCTCTTAAGTTGGAGGAGTGTATT	AATTCTTCTTTATCACAACCCGTGG
IGDCC4 knockout confirmation in mouse	ACCATTCATAACAGCCCTAAAAGGAC	CTCTGCTGGCCTATGATTCCTTCC
IGDCC4 mut-1100YDAI1103-1100FDAV1103	TGTTGCTGCAGGCTCTGGTGTTCGACGCCGTAAAGGGCAATGGG	CCAACACCCGAGACGGCATCAGGAGCCGCTGGAGGCAGGGGGTCCTC
IGDCC4 mut-162EAPDLI1167-1162AAPDAV-1167	GAGGACCCCCTGCCTCCAGCGGCTCCTGATGCCGTCTCGGGTGTTGG	CGCCTGGCTGGGCGTCCGGGAAGGAGCAGGAAGCACTGGCTGCCT
IGDCC4 mut-1210YPDL1213-1210FPDA1213	AGGCAGCCAGTGCTTCCTGCTCCTTCCCGGACGCCCAGCCAGGCG	CCATTTGCGGCCGCGCTAGCCTCGAGGGCAGAGGAGGAGACCGGG

a, The sequences of gRNAs are shown in italic and bold, and the sequences mutated are underlined.

### Plasmids

The cDNA of IGDCC4 was cloned into the mammalian expression vector pCAGGS. The open reading frames (ORFs) of the HA, NA, M1, and M2 genes of H5N1 virus, and the ORFs of the HA genes of H1N1 and H9N2 viruses with a Flag tag at the C-terminus were individually cloned into pCAGGS. IGDCC4, IGDCC4-extracellular, and IGDCC4-cytoplasmic domain with a Myc tag at the C-terminus were cloned into pCAGGS. All plasmid constructs were confirmed by sequencing.

### Antibodies

The commercially obtained primary antibodies used in this study were as follows: GAPDH Rabbit monoclonal antibody (Cell Signaling Technologies, Cat.no. 2118), IGDCC4 Mouse monoclonal antibody (Santa Cruz Biotech, Cat.no. sc-100280), Flag-tag Rabbit monoclonal antibody (Cell Signaling Technologies, Cat.no. 14793), Flag-tag Mouse monoclonal antibody (Cell Signaling Technologies, Cat.no. 8146), c-Myc Rabbit monoclonal antibody (Sigma Aldrich, Cat.no. C3956), and c-Myc Mouse monoclonal antibody (Sigma Aldrich, Cat.no. M4439). The rabbit anti-NP polyclonal antibody was made and stored in our laboratory. Alexa Fluor 633 goat anti-rabbit IgG(H+L) (Thermo Scientific, cat.no. A-21070) and Alexa Fluor 488 goat anti-mouse IgG(H+L) (Thermo Scientific, cat.no. A-11029) were used as secondary antibodies for confocal microscopy. The secondary antibodies used for Western blotting were Odyssey DyLight 800-labeled antibody to Rabbit IgG (H+L) (KPL, Cat.no. 5230–0411) and Odyssey DyLight 800-labeled antibody to Mouse IgG (H+L) (KPL, Cat.no. 5230–0347).

### A549-GeCKO library generation and host factor screening for H5N1 virus infection

The A549-GeCKO library was generated by using the lentiGuide-Puro (Addgene, Cat.no. #52963) two-vector system for Cas9 and sgRNA delivery as previously described [55]. Briefly, 3.4 x 10^8^ A549-Cas9 cells were transduced with lentivirus containing the human sgRNA library (Addgene, Human GeCKO v2 Library, Cat.no. #1000000049) at an MOI of 0.3 to attain no more than 1 sgRNA per cell and at least 1000 cells for each sgRNA. The cells were selected with 1.5 μg/ml puromycin for 14 days to achieve >95% gene disruption. First, 1.2 x 10^7^ A549-GeCKO library cells were subjected to deep sequencing to confirm their library integrity. Then, 1.2 x 10^8^ A549-GeCKO library cells were infected with H5N1 virus at an MOI of 3. Forty-eight hours later, the surviving cells were collected and reseeded to expand up to 1.2 x 10^7^ cells, and were then infected with H5N1 virus at an MOI of 3. This process was repeated one more time, and then the genomic DNA (gDNA) was extracted from the surviving cells by using Quick-gDNA MidiPrep (Zymo 344 Research, Cat. no. D3100) according to the manufacturer’s specifications. The inserted gRNA cassette was amplified by using a Q5 super-fidelity DNA polymerase (New England BioLabs, Cat.no. M0491) as previously described [56]. PCR fragments were assessed with an Agilent 2100 Bioanalyzer and sequenced on an Illumina HiSeq2000 platform (Shenzhen Zhong Nong Jing Yue Biotech Company Limited, China). Raw data were processed first with the fastx toolkit to eliminate reads of low quality and < 50 nt. The perfect reads with the sgRNA 5’ and 3’ flanks were selected and summed with a Perl script as described previously [44]. sgRNA enrichment was ranked and the top 10 of the corresponding genes were identified as candidate hits based on the enriched reads number. Raw data on sgRNA deep sequencing were uploaded to the cloud Sequence Read Archive (SRA) of NCBI (accession number: PRJNA777037).

### Generation of CRISPR KO Cells

Non-targeting control gRNA and gRNAs targeting SLC35A1, IGDCC4, ZFAT, PACSIN2, and ZNF471 were cloned into the plasmid lentiGuide-puro individually and used to generate the lentivirus non-targeting control A549 cells (A549-NT) and polyclonal CRISPR KO cells as previously described [55]. IGDCC4-KO cells were purified from the polyclonal IGDCC4-KO cells by limiting dilution, and knockout of IGDCC4 was confirmed by sequencing and Western blot analyses. Cell plasma membrane protein was isolated from 1×10^7^cells by using the Minute plasma membrane protein isolation and cell fractionation kit (Invent Biotechnologies, cat.no. SM-005) by following the manufacturer’s instructions. The pellet containing plasma membrane proteins were collected to analyze IGDCC4 expression by western blotting. Na+/K+ ATPase was used as a plasma membrane loading control.

### Effect of siRNA knockdown of IGDCC4 on influenza virus replication

siRNA targeting IGDCC4 or scrambled siRNA at a concentration of 40 nM was transfected into A549 cells seeded into 12-well plates by using the Lipofectamine RNAiMAX transfection reagent. The mRNA level of the IGDCC4 was measured by qRT-PCR at 36 h post-transfection to evaluate the knockdown efficiency. The siRNA-treated A549 cells were infected at 36 h post-transfection with H5N1 virus at an MOI of 0.01, and supernatants were collected at the indicated timepoints for virus titration by performing plaque assays in MDCK cells.

### Virus replication in different cells

The polyclonal CRISPR KO cells and A549 cells were infected with H5N1 virus at an MOI of 0.01. Supernatants were collected at 48 h p.i. for virus titration in chicken embryos.

The IGDCC4-KO cells and A549 cells were infected with H5N1, H1N1, or H9N2 at the indicated MOI, and supernatants of H5N1, H1N1 and H9N2 were collected at the indicated timepoints for viral titration in chicken embryos or MDCK cells.

### Cell viability assay

Cell viability was determined by using the CellTiter-Glo kit (Promega, Cat.no. G9242) as described previously [57]. Briefly, IGDCC4-KO and A549 cells were seeded in opaque-walled 96-well plates. Then, 100 μl of CellTiter-Glo reagent was added directly into each well and incubated with the cells for 10 min on a shaker to induce cell lysis. The luminescence was measured with a GloMax 96 Microplate Luminometer (Promega).

### Colocalization analysis by using confocal microscopy

IGDCC4-KO and A549 cells were seeded in glass-bottom culture dishes, infected with H5N1 virus at an MOI of 5, incubated at 4°C for 1 h, and then moved to a 37°C incubator. After being incubated at 37°C for 1 or 2 h, the cells were fixed with 4% paraformaldehyde (PFA) for 15 min, permeabilized with 0.5% Triton X-100 in PBS for 15 min, blocked with 5% BSA in PBS for 1 h, and then incubated with rabbit anti-NP polyclonal antibody and mouse anti-IGDCC4 monoclonal antibody at 4°C overnight. The cells were then washed three times with PBS, and incubated with Alexa Fluor 633 goat anti-rabbit IgG(H+L) and Alexa Fluor 488 goat anti-mouse IgG(H+L) for 1 h. After three washes, the cells were incubated with DAPI (Beyotime Biotech, Cat.no. C1006) for 15 min to stain the nuclei. Images were acquired by using the LSM 800 confocal microscope with Airyscan (Zeiss, Oberkochen, Germany).

To investigate the co-localization of IGDCC4 with HA, NA, or M2, A549 cells were transfected with a Myc-tagged IGDCC4 expressing plasmid (pCAGGS-Myc-tagged IGDCC4) together with a plasmid bearing Flag-tagged HA (pCAGGS-Flag-tagged HA), NA (pCAGGS-Flag-tagged NA), or M2 (pCAGGS-Flag-tagged M2) of H5N1 virus. After being incubated at 37°C for 24 h, the cells were fixed with 4% paraformaldehyde (PFA) for 15 min, permeabilized with 0.5% Triton X-100 in PBS for 15 min, blocked with 5% BSA in PBS for 1 h, and then incubated with rabbit anti-Flag monoclonal antibody and mouse anti-Myc monoclonal antibody at 4°C overnight. The cells were then washed three times with PBS, and incubated with Alexa Fluor 633 goat anti-rabbit IgG(H+L) and Alexa Fluor 488 goat anti-mouse IgG(H+L) for 1 h. After three washes, the cells were incubated with DAPI for 15 min to stain the nuclei. Images were acquired by using the LSM 800 confocal microscope with Airyscan.

### Analysis of sialic acid receptors

To analyze sialic acid receptors, IGDCC4-KO and A549 cells were, respectively, seeded in 10-cm dishes. After 24 h, the cells were treated with trypsin, counted, fixed with 4% PFA for 15 min, and then stained with Alexa Fluor 647 conjugated with wheat germ agglutinin (WGA) (Thermo Scientific, Cat.no. W32466). A549 cells and IGDCC4-KO cells (negative controls) were pretreated with neuraminidase (1 unit/well) (Sigma-Aldrich, Cat.no. N2876-25U) and incubated at 37°C for 1 h before being treated with trypsin. After three washes with PBS, the cell suspensions were subjected to flow cytometry on a FACSAria flow cytometer (BD Biosciences, Franklin Lakes, NJ). The data were analyzed by using FlowJo software (FlowJo, Ashland, OR).

### Analysis of influenza virus attachment and internalization

To analyze the effect of IGDCC4 on influenza virus attachment to the cell surface, IGDCC4-KO and A549 cells in 12-well plates were infected with H5N1 virus at an MOI of 5 and incubated at 4°C for 1 h. The cells were then washed with ice-cold PBS (pH = 7.2) or ice-cold acidic PBS (pH = 1.5), lysed with 4× SDS-PAGE loading buffer (with β-Mercaptoethanol) (Solarbio, Cat.no. P1016), and subjected to Western blotting with a rabbit anti-NP polyclonal antibody.

Acidic PBS (pH = 1.5) can elute virus particles on the cell surface that have not been internalized into cells [14]. To determine the effect of IGDCC4 on influenza virus internalization, A549 cells, pretreated for 30 minutes at 37°C with or without 80 μM/ml dynasore, and IGDCC4-KO cells in 12-well plates were infected with H5N1 virus at an MOI of 5. After being incubated at 4°C for 1 h and at 37°C for 1 h, the cells were washed three times with ice-cold PBS (pH = 7.2) or ice-cold acidic PBS (pH = 1.5) to remove the attached but not-yet-internalized virions. The cells were then collected and lysed for Western blotting with a rabbit anti-NP polyclonal antibody.

To investigate whether overexpression of IGDCC4 could restore the internalization of influenza virus into IGDCC4-KO cells, we infected A549 cells and IGDCC4-KO cells that were transfected with pCAGGS and IGDCC4-KO cells that were transfected with pCAGGS-IGDCC4 with H5N1 virus at an MOI of 5. After being incubated at 4°C for 1 h and 37°C for 1 h, the cells were washed three times with ice-cold acidic PBS (pH = 1.5) to remove the attached but not-yet-internalized virions. The washed cells were then collected to detect the NP protein level by Western blotting and RNA level by qRT-PCR.

To investigate the attachment of VSV-GFP, A549 cells and IGDCC4-KO cells infected with VSV-GFP virus at an MOI of 10 were incubated at 4°C for 1 h and were then washed three times with ice-cold PBS (pH = 7.2). To investigate the internalization of VSV-GFP, the cells were further incubated at 37°C for 1 h after being incubated at 4°C for 1 h, and were then washed three times with ice-cold acidic PBS (pH = 1.5) to remove the attached but not-yet-internalized virions to detect internalization of VSV-GFP virus. Viral RNA level of these treated cells was measured by qRT-PCR.

### qRT-PCR

To analyze viral NP expression, total RNA of virus infected cells was extracted by using a RNeasy kit. Relative quantities of viral NP genomic RNA (vRNA) and mRNA were determined by qRT-PCR as described previously [30]. Relative RNA quantities were determined with GAPDH as the endogenous reference. The primer sequences are listed in Table 1.

### Co-immunoprecipitation assay

Co-immunoprecipitation assay was performed as described previously [58]. In brief, HEK293T cells grown in 6-well plates were transfected with the indicated plasmids by using Lipofectamine LTX and Plus reagents (Invitrogen, Cat.no. 15338100). At 36 h post-transfection, the cells were washed once with ice-cold PBS (pH = 7.2) and lysed with Pierce IP Lysis Buffer (Thermo Scientific, Cat.no. 87788) containing a complete protease inhibitor cocktail (Abcam, Cat.no. ab65621) and phenylmethylsulfonyl fluoride (PMSF) (Sigma-Aldrich, Cat.no. 10837091001) for 1 h on ice. The supernatants were then incubated with a mouse anti-Flag monoclonal antibody or a mouse anti-Myc monoclonal antibody and protein G-agarose (Roche, Cat.no. 11243233001), and rocked at 4°C overnight. The beads were then washed three times with ice-cold PBS, and the bound proteins were separated by SDS-PAGE and detected by Western blotting.

### Western blotting

Protein samples fractionated by SDS-PAGE were transferred onto nitrocellulose membranes (GE Healthcare, Cat.no. 10600002). Membranes blocked with 5% skim milk in PBS were incubated overnight at 4°C with the appropriately diluted primary antibodies in Primary Antibody Dilution Buffer (Beyotime Biotech, Cat.no. P0023A). After incubation with Odyssey DyLight 800-labeled Antibody to Rabbit IgG (H+L) or Odyssey DyLight 800-labeled Antibody to Mouse IgG (H+L), blots were visualized by using an Odyssey CLX infrared imaging system (Li-Cor BioSciences, Lincoln, NE).

### Transferrin internalization assay

The medium of IGDCC4-KO and A549 cells was replaced with 1 mL of Opti-MEM. After incubation for 1 h at 37°C, the cells were treated with 50 μg/ml Alexa 488-labeled transferrin (Thermo Scientific, Cat.no. T13342) for 30 min at 37°C. After being washed three times with acid strip buffer (50 mM glycine, 100 mM NaCl, pH = 3.0), the cells were incubated with DAPI for 15 min to stain the nuclei. Images were acquired by using the LSM 800 confocal microscope with Airyscanas. Automated image analysis was used to quantify the transferrin fluorescence intensity from 100 cells.

### Animal study

*IGDCC4*^*−/−*^ mice were generated by BIOCYTOGEN, Inc (Jiangsu, China). Genotyping to distinguish wild-type from knockout mice was performed by use of PCR with the primers listed in Table 1. All mice had the C57BL/6J background and were maintained under specific-pathogen-free conditions.

Groups of 13 wild-type and *IGDCC4*^*−/−*^ mice were intranasally infected with five 50% mouse lethal dose (MLD_50_) of H5N1 virus. Three mice in each group were euthanized on day 3 post-infection and their organs were collected for virus titration in eggs. The other 10 mice in each group were monitored daily for body weight changes and survival for a total of 14 days. The lethality test was repeated once with 10 mice in each group.

### Statistical analysis

Quantitative data are presented as means ± standard deviations (SDs) of three independent experiments or replicates. Data were statistically analyzed by using the Student’s two-tailed unpaired t test with GraphPad Prism software (GraphPad, San Diego, CA). Statistical parameters are reported in the figures and figure legends. *P* values < 0.05 were considered statistically significant.

## Supporting information

S1 Table sgRNAs and genes enriched in each CRISPR/Cas9 screen for highly pathogenic H5N1 virus infection. (XLSX)

S1 Fig Effect of H5N1 virus infection on parental A549 and A549-NT cells. (A) Viability of A549-NT cells was measured by using the CellTiter-Glo assay and compared with that of A549 cells. (B) Replication of H5N1 virus in A549 and A549-NT cells. A549 and A549-NT cells were infected with H5N1 virus at an MOI of 0.01. Supernatants were collected at the indicated timepoints for virus titration in MDCK cells. The data shown are from three replicates (means ± SDs).
(TIF)

S2 Fig Overexpression of IGDCC4 in HEK293T cells. HEK293T cells were transfected plasmids for the expression of IGDCC4 fused with a Myc tag. Western blot analysis showed that both the isoform-1 (about 240 kDa) and the isoform 2 (about 130 kDa) were detected.
(TIF)

S3 Fig Mutation of the endocytic signals in the cytosolic domain of IGDCC4 does not affect the internalization of influenza virus. (**A**) Schematic illustration of the endocytosis signals in the cytoplasmic domain of IGDCC4. (**B**) Schematic illustration of the mutations made in the endocytic signals in the cytoplasmic domain of IGDCC4. The numbers indicate the starting and ending positions of the signals. The amino acids are represented by the single-letter code, X indicates any amino acid, Ø indicates an amino acid with a bulky hydrophobic side chain, and the brackets mean that either amino acid is allowed at that position. The mutated amino acids are underlined in panel B. (**C**) Overexpression of IGDCC4 and its mutants restores the internalization of influenza virus into IGDCC4-KO cells. The protein level of IGDCC4 and NP in the A549 cells transfected with different IGDCC4 constructs was determined by Western blotting. (**D**) The mRNA level of IGDCC4 in the A549 cells transfected with different constructs was determined by qRT-PCR and standardized to that in the pCAGGS-transfected cells. (**E**) The RNA level confirmed that overexpression of IGDCC4 and IGDCC4-mut restores the internalization of influenza virus into IGDCC4-KO cells. The data shown are from three independent experiments or replicates (means ± SDs). Of note, the mRNA level of IGDCC4 and IGDCC4-mut was comparable, but the IGDCC4 was not blotted probably because these mutations resulted in the loss of epitopes recognized by the monoclonal antibodies used. The two-tailed unpaired t-test was used for the statistical analysis. ns denotes non-significant.
(TIF)
