# Supplementary figures and images for: A genome-wide CRISPR/Cas9 gene knockout screen identifies immunoglobulin superfamily DCC subclass member 4 as a key host factor that promotes influenza virus endocytosis
Source: PLoS Pathog. 2021 Dec 6;17(12):e1010141. doi: 10.1371/journal.ppat.1010141 (PMC8675923; doi:10.1371/journal.ppat.1010141)

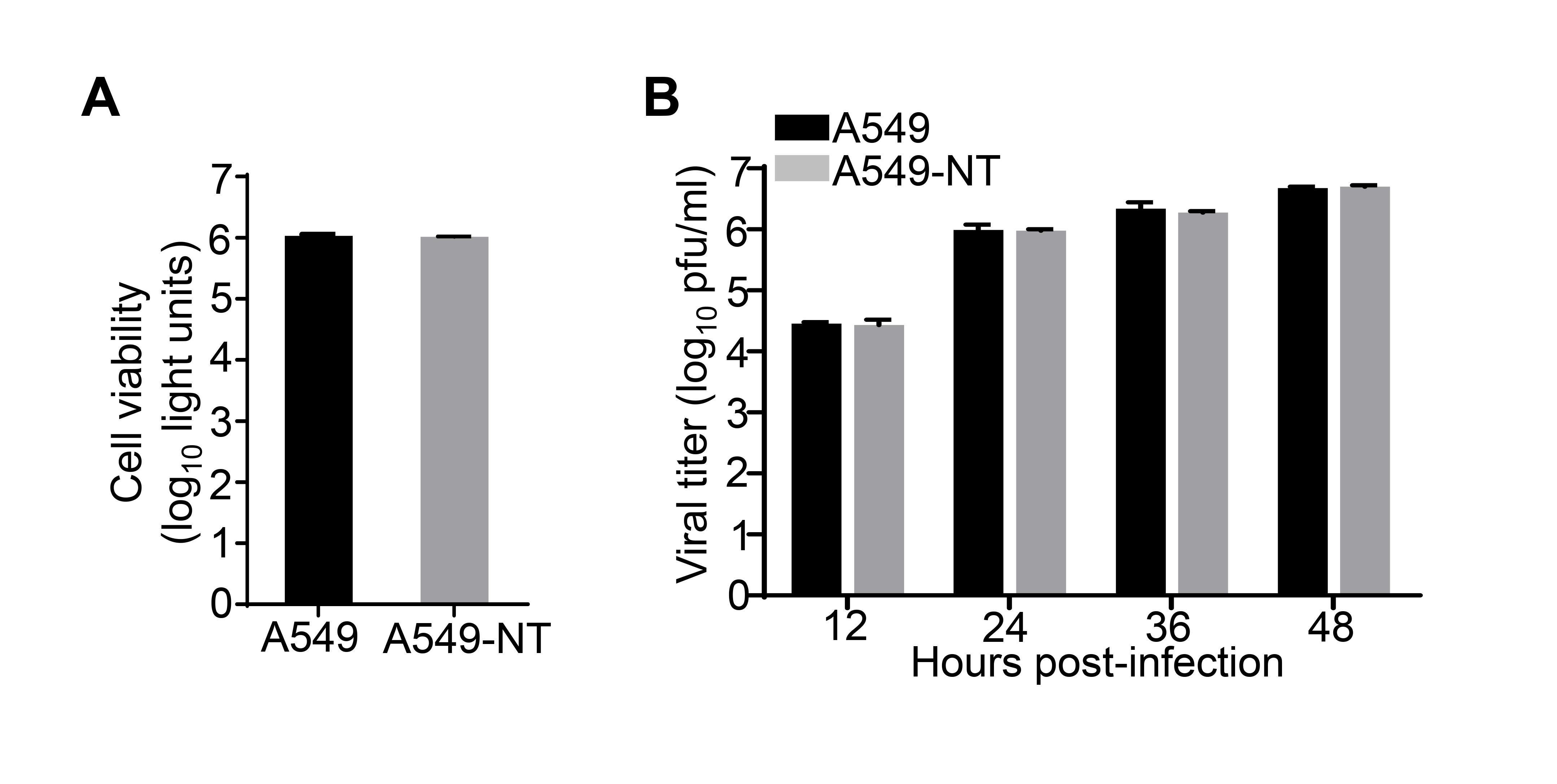

Supplement: S1 Fig — (A) Viability of A549-NT cells was measured by using the CellTiter-Glo assay and compared with that of A549 cells. (B) Replication of H5N1 virus in A549 and A549-NT cells. A549 and A549-NT cells were infected with H5N1 virus at an MOI of 0.01. Supernatants were collected at the indicated timepoints for virus titration in MDCK cells. The data shown are from three replicates (means ± SDs). (TIF) [file ppat.1010141.s002.tif]

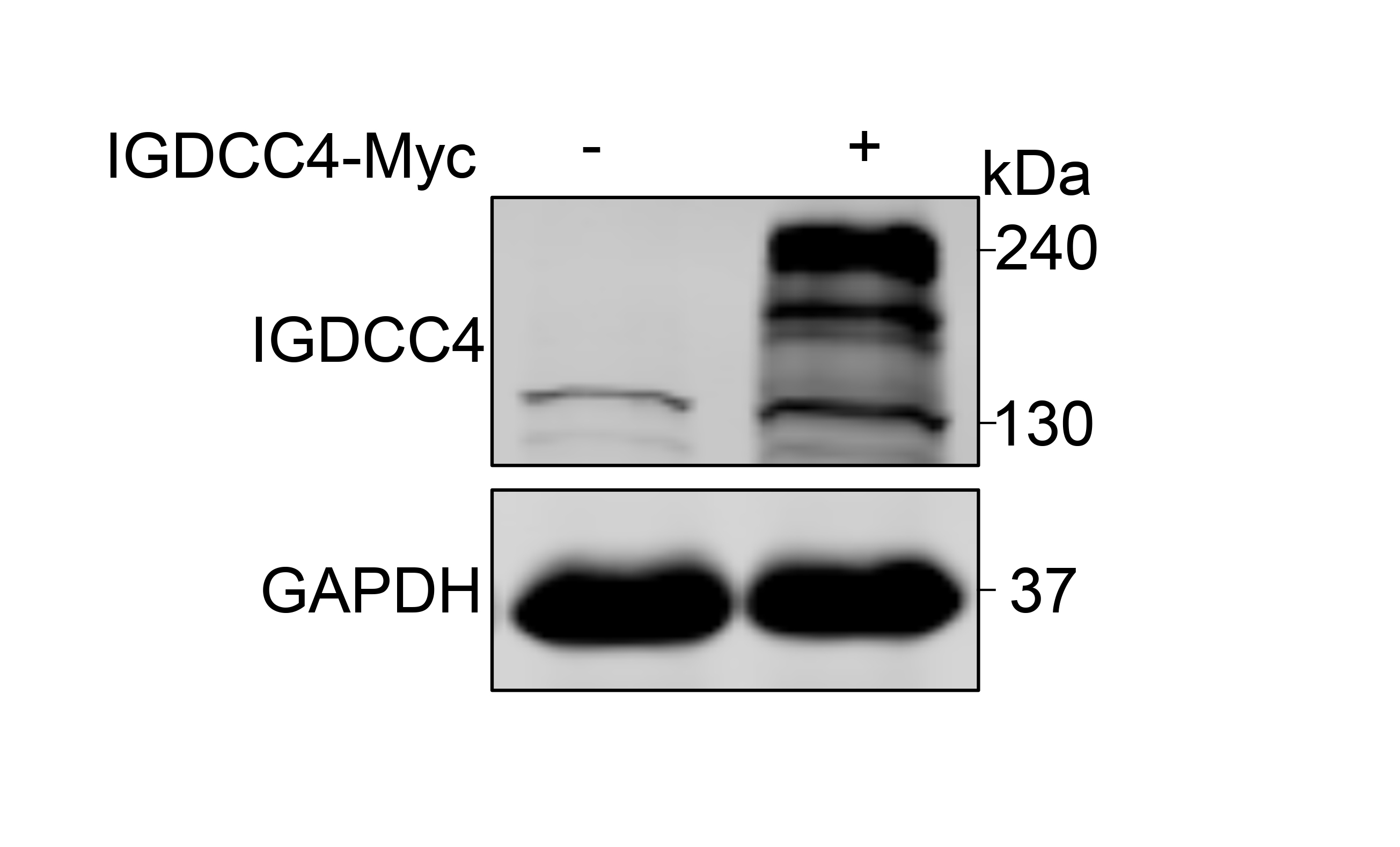

Supplement: S2 Fig — HEK293T cells were transfected plasmids for the expression of IGDCC4 fused with a Myc tag. Western blot analysis showed that both the isoform-1 (about 240 kDa) and the isoform 2 (about 130 kDa) were detected. (TIF) [file ppat.1010141.s003.tif]

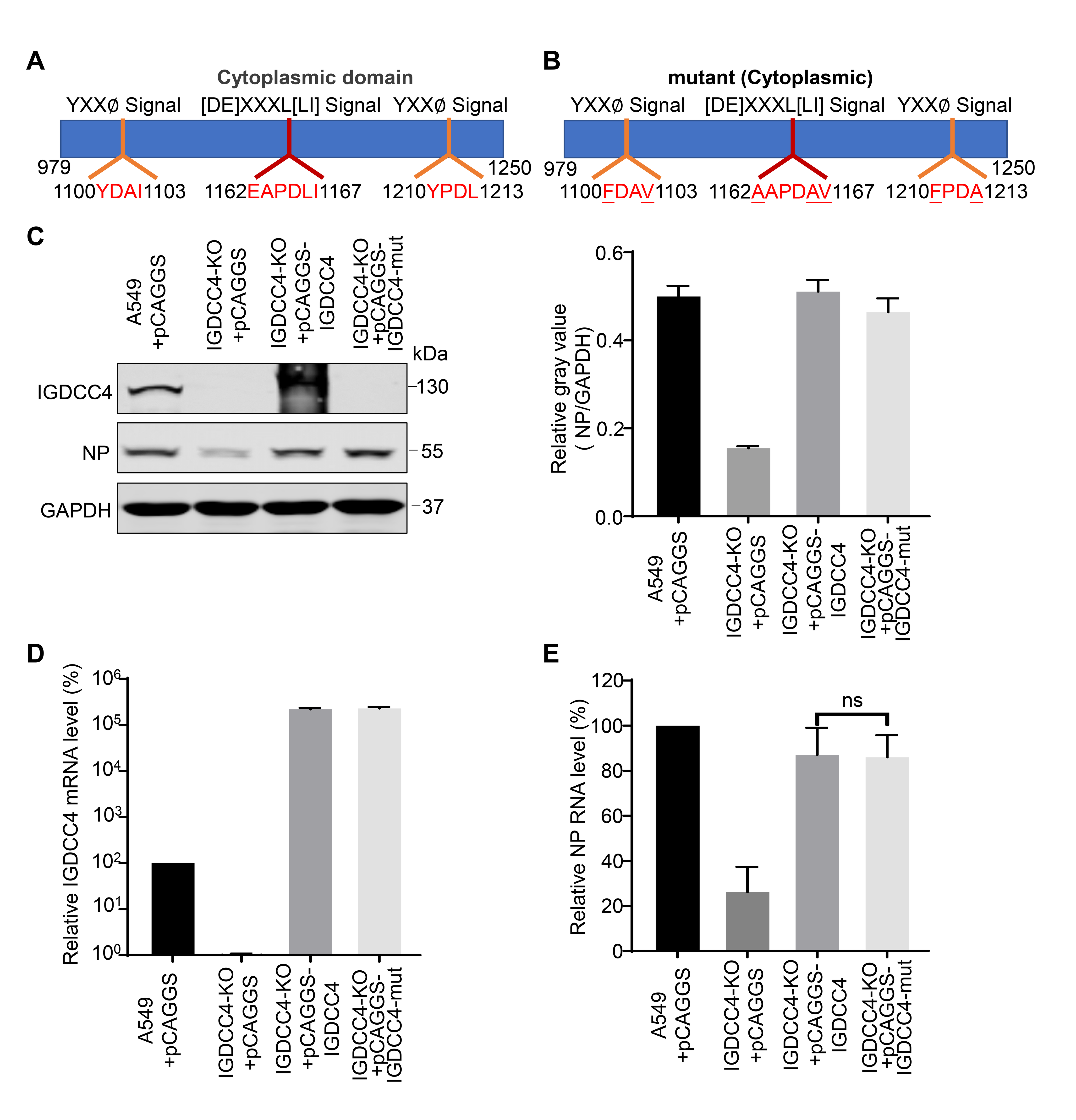

Supplement: S3 Fig — (A) Schematic illustration of the endocytosis signals in the cytoplasmic domain of IGDCC4. (B) Schematic illustration of the mutations made in the endocytic signals in the cytoplasmic domain of IGDCC4. The numbers indicate the starting and ending positions of the signals. The amino acids are represented by the single-letter code, X indicates any amino acid, Ø indicates an amino acid with a bulky hydrophobic side chain, and the brackets mean that either amino acid is allowed at that position. The mutated amino acids are underlined in panel B. (C) Overexpression of IGDCC4 and its mutants restores the internalization of influenza virus into IGDCC4-KO cells. The protein level of IGDCC4 and NP in the A549 cells transfected with different IGDCC4 constructs was determined by Western blotting. (D) The mRNA level of IGDCC4 in the A549 cells transfected with different constructs was determined by qRT-PCR and standardized to that in the pCAGGS-transfected cells. (E) The RNA level confirmed that overexpression of IGDCC4 and IGDCC4-mut restores the internalization of influenza virus into IGDCC4-KO cells. The data shown are from three independent experiments or replicates (means ± SDs). Of note, the mRNA level of IGDCC4 and IGDCC4-mut was comparable, but the IGDCC4 was not blotted probably because these mutations resulted in the loss of epitopes recognized by the monoclonal antibodies used. The two-tailed unpaired t-test was used for the statistical analysis. ns denotes non-significant. (TIF) [file ppat.1010141.s004.tif]
